# ''United Airways Disease'' and Phenotypic Peculiarities of Respiratory Allergy in Immigrants

**DOI:** 10.1186/1939-4551-2-2-13

**Published:** 2009-02-15

**Authors:** Carlo Lombardi, Giovanni Passalaqua, Giorgio Walter Canonica

**Affiliations:** 1 The Allergy and Respiratory Diseases, Department of Internal Medicine, University of Genoa, Genoa, Italy

**Keywords:** asthma, rhinitis, allergy, immigration, migrants

## Abstract

Allergy is the result of a complex interaction between genetic background and environmental factors, including exposure to allergens and lifestyle. Migration is a process that involves many radical changes in the environment, including diet, pollutants, allergens, different housing conditions, and patterns of infections. Thus, studies in immigrants may provide important information about the role of environmental factors in the development of allergic respiratory diseases. Several studies addressed this aspect and consistently found that migrants develop allergies at different rates from the local population, and very often the symptoms appear with a delay of 3 to 5 years after migration. More recent data showed that the severity of allergic diseases is greater in migrants, and that usually the onset is with associated asthma and rhinitis. The immigration model strongly suggests that environmental factors overcome the genetic background, and that the clinical phenotype of respiratory allergy in migrants has some peculiarities.

## 

Respiratory allergy (ie, rhinitis and asthma) has a high prevalence and, at least for rhinitis, the prevalence is constantly increasing worldwide. Currently, the accepted theory to explain the increasing prevalence of allergy is the so-called *hygiene hypothesis *[1] based on the supposition that the decreased infectious burden in infancy (caused by the improved lifestyle) may favor a T-helper cell 2 (Th2)-oriented immune response. In fact, it is well known that bacterial, fungal, and viral cell wall and nucleic acid products are capable of stimulating Toll receptors, pertaining to the innate immune response, and to orient the acquired immunity toward a Th1 pathway[2].

Nonetheless, things are probably more complex because both genetic and environmental determinants intervene in the development of atopy[3]. In this sense, it is unlikely that a change in the genetic background could account for the increasing trend in prevalence, whereas environmental conditions (including pollution, infections, food) are probably more important (Figure 1)[4,5].

**Figure 1:**
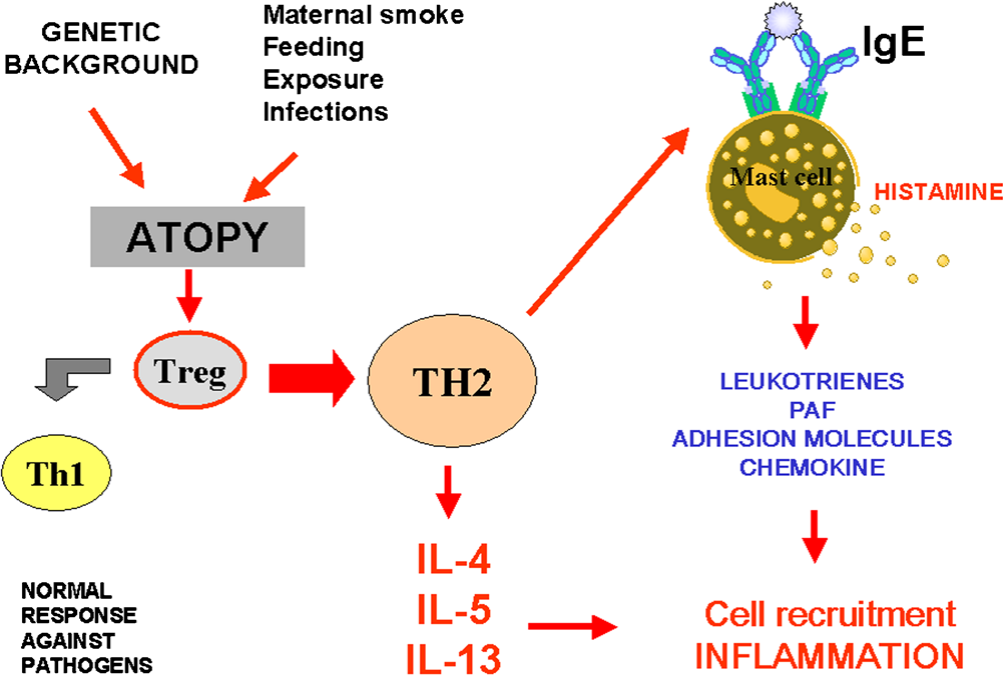
**The overall scheme of the development of atopy and subsequent allergic inflammation**.

The hygiene hypothesis envisages the possibility of preventing allergic respiratory diseases since infancy. In fact, a susceptible genetic background with specific environmental stimuli will lead to the development of asthma in children. Conversely, sufficiently low environmental stimuli would result in a protection against the development of allergic diseases in susceptible genotypes. The hygiene hypothesis suggests that development of allergy (or asthma) can be prevented by a shift from Th2 predominance to Th1, induced by exposure to immune stimulants such as viruses, bacteria, and endotoxins, in particular in the prenatal period or early childhood (Figure 1)[6]. In humans who are predisposed to atopy, immune recognition results in production of an allergic response typified by proliferation of Th2-type T lymphocytes. This Th2-driven process is the defining immunopathologic characteristics of respiratory allergic diseases such as asthma and rhinitis[7].

## Allergic respiratory diseases in migrants: an emerging problem and an epidemiological model

Respiratory allergy is one of the clinical manifestations of the ''atopic status,'' which is characterized by an ongoing production of specific immunoglobulin E and which is the result of a complex interaction among genetic and environmental factors[1,8]. One of the effects of the environment in a broad sense can be grossly appreciated in the variations of prevalence that is higher in developed and high-income countries than in developing and low-income countries[9].

Migration is a process that involves many cultural and socioeconomic issues such as the changes in lifestyle, the effects of new diet schedules, the contact with new pollutants and allergens, the different housing conditions and patterns of infections, and usually a better accessibility to medical resources[10]. Thus, studies in immigrants may provide important information about the role of environmental factors in the development of allergic respiratory diseases[11].

Previous studies have focused on the differences among ethnic groups and have suggested that asthma prevalence and mortality in the United States are lower in Mexicans than in other Hispanic ethnicities or in African Americans[12]. Noteworthy, the differences in asthma rates were found to be dependent not only on the birthplace, but also on the duration of the stay in the United States. In addition, other studies from Australia, Europe, and Israel have documented the importance of immigration and acculturation in the development of allergic respiratory diseases. In an Australian cross-sectional study, the prevalence of wheeze or asthma was higher in Australian-born non-Asians and Australian-born Asians than in Asian immigrants of Chinese origin[13]. In contrast, regardless of where they were born, hay fever prevalence was higher in Asian immigrants than in Australian-born non-Asians. An important point was that the prevalence of hay fever and asthma in Asian immigrants increased with length of stay in Australia. Another significant finding was that the new environment influenced the type of allergen sensitization. Among the Asian immigrants to Melbourne, an increased prevalence of pollen and mite allergy correlated well with the increased exposure to those allergens after migration, whereas these specific allergies were less frequent in subjects living in China, Hong Kong, and other areas of Southeast Asia[14].

Mexican American children born in the United States had higher rates of sensitization to allergens that are well represented in the United States, including cat, house-dust mite, *Alternaria*, peanut, and typical United States pollens. In contrast, they displayed a lower prevalence of sensitization to cockroach (23% for Mexican American children born in the United States compared with 40% for Mexican American children born in Mexico)[12].

A study conducted in extra-European immigrants in Milan showed that allergy and asthma symptoms developed usually only after immigration[15]. The patients were complaining of asthma (63.7%), rhinoconjunctivitis (56.7%), or rhinitis alone (21%). Among them, 84.3% declared that they were healthy before migration, and allergic respiratory diseases started 4 months to 7 years after their arrival in Italy. Another cross-sectional Italian study assessed the prevalence of allergic respiratory diseases and sensitizations in Albanian migrants to southern Italy[16]. This study demonstrated that Albanian migrants, despite the low prevalence of allergic diseases and sensitization in their country of origin, developed an increasing prevalence of sensitization to local allergens and nasal symptoms (15.8%) after migration.

A large cross-sectional study conducted in migrant children to Italy (SIDRIA-2) compared the prevalence of respiratory symptoms in 29,305 migrant and nonmigrant children residents in Italy and examined the effect of the duration of stay in Italy. Migrant children who had lived in Italy for 5 years or longer had lifetime asthma and current wheeze risks close to the Italian children[17]. A study conducted in Israel evaluated the trends in specific morbidity prevalence in male adolescents during a 50-year period and the impact of recent immigration[18]. The prevalence of asthma increased dramatically from 1.2% in 1957-1961 to 11.1% in 2003-2004, and the patterns of disease prevalence were different for immigrants.

## "Immigrant-United Airways Disease": A new phenotype of respiratory allergy?

In a recent survey, we collected the data of 237 adult immigrants (129 women, 108 men; mean age, 36.4 years; age range, 18-54 years) living in Brescia, Northwest Italy[19]. The data were compared with a matched sample of the local population. Brescia has a heavy immigration burden because of the presence of numerous manufactures and farms. In 2005, it was estimated that about 130,000 migrants lived in the district; 30,000 of them within the town, this corresponding to about 12% of the total population[20]. We assessed the demographic and clinical characteristics of the immigrants who were seen at the emergency care unit of our hospital and/or referred to our unit for respiratory allergic diseases from the period 2002 to 2007. Their macroregion of origin were distributed as follows: Africa, 31%; eastern Europe, 24%; Asia, 23%; and South America, 22%. As assessed by the medical history, symptoms appeared for the first time between 4 and 7 years (mean, 5.21 years) after the arrival to Italy. Surprisingly, only 22 (9%) of 237 patients had a positive family history for atopy, and only 5 (2%) of 237 of the subjects had a positive clinical history for rhinitis and/or asthma in the past. These percentages substantially differed from those seen in the local population (63% and 45%, respectively). About 60% of the immigrants had concomitant rhinitis and asthma at the onset, whereas this percentage was 40% in the local population. Rhinitis, but not asthma, was overall more severe in immigrants. All had positive skin prick tests to at least one of the common aeroallergens, but only 25.3% were monosensitized, whereas the remaining patients were polysensitized to 2 or more aeroallergens. Interestingly, the rate of skin positivity to cockroach was found to be higher (20%) in immigrants than in the native population (< 5%). In addition, some of the patients were sensitized to plants (eg, *Parietaria*) that are not present in the region of origin.

Based on these findings, it seems that the clinical phenotype of respiratory allergy in immigrants shows some peculiarities, such as the absence of previous clinical history and familiar history, and the overall greater severity at the onset. In particular, in most of the immigrants, the respiratory allergy appeared since the beginning as ''United Airways Disease,'' with concomitant rhinitis and asthma.

## Conclusiogns

Immigrants are a particular population, as they undergo a sudden and permanent change in lifestyle/environment, and can therefore represent an interesting epidemiological model to evaluate the impact of environmental factors on diseases because a genetic variation to explain the onset of respiratory allergy can be reasonably excluded.

Migrants undergo exposure to new pollutants and allergens and come into contact with new socioeconomic and cultural aspects such as housing conditions, diet, and medical services. We should hypothesize that even in nonpredisposed subjects, the new environmental factors can activate, within a few years, the immune system to develop an atopic response to environmental proteins. It is true that the higher prevalence of skin positivity to cockroach, which is not typical of industrialized countries, could suggest a previously acquired sensitization probably caused by the more crowded environments in the regions of origin. This is only a hypothesis and should be confirmed by skin testing immigrants upon arrival.

In summary, our data and many other evidences in migrants support the idea that: (1) lifestyle and environmental factors in western industrialized countries facilitate the development of allergic respiratory diseases; (2) immigration to allergy-prevalent countries causes more allergic respiratory diseases in immigrants as compared with the prevalence of atopy in their countries of origin; (3) the increase in allergic respiratory diseases is usually not related to ethnicity; (4) the effect is time dependent; (5) and immigrants are more prone to develop allergies than local population and display some phenotypic characteristics (Table 1).

**Table 1:** Differences Between Immigrant and Nonmigrant-Resident Allergic Respiratory Diseases That Suggest the Possibility of Distinct ''United Airways Disease'' Phenotype

Parameter	Immigrants	Nonmigrants/Residents
Severity grade of allergic respiratory diseases	Moderate-high	Variable
Rhinitis and asthma comorbidity	Very high	Moderate-high
Allergen polysensitization-monosensitization ratio	High	Moderate
Cockroach sensitization (Italy data)	High	Low
Drug and/or food allergy	Low	Moderate-high
Compliance to therapy	Low	Moderate-high
Previous familial history of atopy	Low	Moderate-high

Migrants should be aware of the potential of developing allergies and/or rhinitis and asthma. Plans of action for primary prevention in high-risk atopic individuals and secondary prevention protocols should be developed both for populations in developing countries as well as for immigrants from such countries to developed and industrialized atopy-prevalent countries. Carefully designed cross-sectional national and geographically specific surveys might provide additional insight into the relationship between immigration, acculturation, and the risks of allergy and asthma.

## End Note

This work has been partially supported by the Associazione Ricerca Malattie Immunologiche e Allergiche
